# Tilted pulse front pumping techniques for efficient terahertz pulse generation

**DOI:** 10.1038/s41377-023-01293-1

**Published:** 2023-10-24

**Authors:** György Tóth, Gyula Polónyi, János Hebling

**Affiliations:** 1 University of Pécs, Pécs, 7624 Hungary; 2 Szentágothai Research Centre, Pécs, 7624 Hungary; 3 HUN-REN-PTE High-Field Terahertz Research Group, Pécs, 7624 Hungary

**Keywords:** Terahertz optics, Optical materials and structures

## Abstract

Optical rectification of femtosecond laser pulses has emerged as the dominant technique for generating single- and few-cycle terahertz (THz) pulses. The advent of the tilted pulse front pumping (TPFP) velocity matching technique, proposed and implemented two decades ago, has ushered in significant advancements of these THz sources, which are pivotal in the realm of THz pump-probe and material control experiments, which need THz pulses with microjoule energies and several hundred kV/cm electric field strengths. Furthermore, these THz sources are poised to play a crucial role in the realization of THz-driven particle accelerators, necessitating millijoule-level pulses with tens of MV/cm electric field strengths. TPFP has enabled the efficient velocity matching in lithium niobate crystals renowned for their extraordinary high nonlinear coefficient. Moreover, its adaptation to semiconductor THz sources has resulted in a two-hundred-times enhancement in conversion efficiency. In this comprehensive review, we present the seminal achievements of the past two decades. We expound on the conventional TPFP setup, delineate its scaling limits, and elucidate the novel generation TPFP configurations proposed to surmount these constraints, accompanied by their preliminary outcomes. Additionally, we provide an in-depth analysis of the THz absorption, refractive index, and nonlinear coefficient spectra of lithium niobate and widely used semiconductors employed as THz generators, which dictate their suitability as THz sources. We underscore the far-reaching advantages of tilted pulse front pumping, not only for LN and semiconductor-based THz sources but also for selected organic crystal-based sources and Yb-laser-pumped GaP sources, previously regarded as velocity-matched in the literature.

## Abstract (web summary)

We review the two decades of tilted pulse front pumping, and suggest its applications to new materials and laser sources, emphasizing its potential with semiconductors and organic crystals for promising advancements.

## Graphical abstract

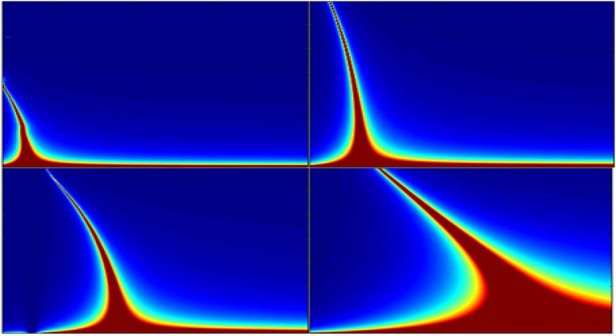

## Introduction

Ultrashort terahertz (THz) pulses with sub-pJ energy and 100 V/cm electric field are widely used for time-domain THz spectroscopy (TDTS)^1^ and THz imaging^2^. For more advanced applications such as THz pump-probe and nonlinear spectroscopic measurements, THz pulses with elevated energy and electric field (usually 1 μJ & >100 kV/cm) enable the precise examination of excitation evolution within the sub-ps time range^3,4^. The same pulses are applicable usually for the manipulation of matters, including lattice and molecular excitations and field-free orientation of molecules^5,6^, as well as controlling electron beams, charge-, and spin- waves^7^, and material structures^8^. THz pulses with even higher energy (on the level of mJ) and electric field (on the level of MV/cm) are needed for enhancement of high harmonic generation^9^, acceleration of charged particles^10–13^, and generation of carrier-envelope-phase stable attosecond pulses^14^.

These THz pulses (regardless of their energy) are generated by photoconductive antennas^15^, by laser-induced plasma^16^, in spintronic structures^17^, and by optical rectification in nonlinear crystals^18–22^, all of them driven by ultrashort laser pulses. Photoconductive antennas, mostly used in TDTS devices, generate single-cycle pulses with a spectral bandwidth of 4–6 THz, with pulse energies in the sub-pJ range^15^. In dual-color laser plasmas, THz pulses with even 20 THz broad spectrum can be achieved^16^. In case of pumping at mid-infrared wavelength (3.9 μm) THz pulses with energies up to the sub-mJ-level were generated^23^, although its utilization has not been spread due to the unusual pumping wavelength and the properties of the THz propagation.

Optical rectification (OR, see Ch. 2) is a nonlinear optical frequency conversion process. The time trace of the electric field, the spectrum, and the conversion efficiency of THz pulses generated via OR, besides the pump pulse duration, strongly depend on the properties of the used nonlinear optical crystal. The most important properties are the nonlinear optical coefficient, the optical and THz absorption spectrum, and the optical and THz refractive index spectrum. The last two are important since it determines the fulfillment of phase matching, which is crucial for all the nonlinear frequency conversion processes.

**Fig. 1:**
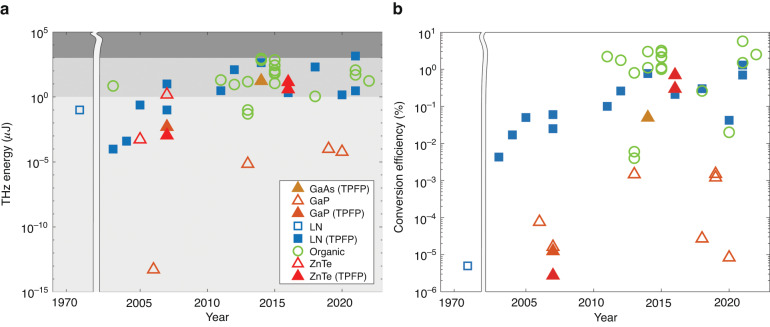
Review of the results from the past 20 years. The energy (**a**) and conversion efficiency (**b**) of broadband THz pulses generated by optical rectification in the last two decades are presented on a logarithmic scale. The filled symbols correspond to TPFP and the empty symbols to collinear setups. The squares are LN, the triangles are semiconductors and the circles are organic crystals. By applying TPFP the conversion efficiency could be increased by several orders for both LN and semiconductor materials. In (**a**), the light grey area corresponds to THz pulses with energy above 1 μJ enabling nonlinear THz applications, while the dark grey area represents THz pulses with energy exceeding 1 mJ, specifically for extreme THz science which has been solely accomplished using LN thus far. A version of these figures with references can be found in the supplementary material

Fig. 1 shows through some typical examples the energies (Fig. 1a) and conversion efficiencies (Fig. 1b) of THz pulse generation by OR in the last 20 years. Based on the used nonlinear material (NM) the following groups were selected: organic crystals (circles), semiconductors (triangles), and lithium niobate (LN) ferroelectric crystal (squares). The filled symbols correspond to tilted pulse front pumping (TPFP), which will be detailed later. As can be seen, for the same material, applying TPFP increases the efficiency by several orders.

Although both the THz energy and conversion efficiency depend on the pumping conditions, primarily of the energy and pulse duration, and in our experience, the measured energy values could differ by a factor of two with different THz energy meters, it seems to be obvious that organic crystals^24–26^ provide the highest efficiencies. At first sight this could be surprising since no special phase matching was applied, only collinear pumping was used. This could be owing to that organic crystals have the largest nonlinear optical coefficients (3–5 times larger than LN, and 6–10 times larger than semiconductors). On the other side, their damage threshold is low (typically around 20 mJ/cm^2^) and they show crystal degradation over prolonged exposure^26^. It is also noteworthy, that these materials require pump sources different from the widespread Ti:sapphire (Ti:s) and Yb-based high energy femtosecond lasers, they provide efficient generation with only special pump sources (with e.g. 1.3–1.7 *μ*m wavelength lasers or with OPA-s), which significantly decreases the wall-plug efficiency.

In case of ZnTe and GaP semiconductors, collinearly phase-matched OR is realized when they are pumped at Ti:s^20^ and Yb lasers^27–30^, respectively. However, since their bandgap are relatively low, the significant 2-photon and 3-photon absorption (2PA - in case of ZnTe, 3PA - in case of GaP) at these wavelengths reduces the available conversion efficiency to around 0.02%^27,29,30^ due to the generation of free carriers which increases the THz absorption^28^. This conversion efficiency can be increased by at least two orders of magnitude when longer pumping wavelength^31–33^ and TPFP^34–36^ are applied. It is important to highlight, that while organic crystals work with high efficiency only at specific wavelengths, semiconductors utilizing TPFP can be pumped at various wavelengths, the only demand is that the pumping wavelength should be long enough to suppress the low-order multiphoton absorptions (MPA).

LN and lithium tantalate (LT) ferroelectric materials have a THz refractive index significantly larger (by more than twice) than the optical refractive index. Hence the phase matching for efficient THz generation was only realized in TPFP scheme ^36,37^. Since these materials own large bandgaps, when pumped by Ti:s lasers there is no 2PA, when pumped by Yb lasers neither 3PA occurs. Moreover, for the large bandgap, the 3PA and 4PA coefficients are much smaller^38^, than in case of semiconductors. For this, the pumping intensity can be significantly larger than in case of semiconductors or organic crystals. For Ti:s laser pumping saturation of the efficiency is predicted^39^ above 30 GW/cm^2^ and for Yb-laser pumping saturation becomes observable^28^ only above 100 GW/cm^2^. Hence the conversion efficiency of THz generation can exceed 1% and the generated pulse energies exceed 1 mJ^40^. At present, LN in TPFP scheme provides the highest energy (see Fig. 1a) and this is the most common OR-based THz source.

In this comprehensive review, we will delve into the remarkable achievements of tilted pulse front pumping over the past two decades. We will also illuminate its inherent limitations while providing a thought-provoking outlook on the promising future possibilities it holds.

## Terahertz pulse generation by optical rectification

Optical rectification is a second-order nonlinear optical process, which was observed first in 1962 when with an approximately 60 ns long ruby laser pulse a static electric field was generated around a KDP crystal^41^. For THz generation, the same nonlinear optical process is applied with much shorter, a few picosecond^42^, or sub-ps pumping laser pulse durations.

Assuming that the Gaussian shape pump pulse does not change significantly during the THz generation process, the

1 $${W(L,\Omega )\propto {\rm{|}}{E}_{{THz}}\left(L,\Omega \right){\rm{|}}}^{2}=\frac{{\Omega }^{2}{d}_{{eff}}^{2}{I}_{0}^{2}{\tau }^{2}{L}^{2}}{4{\rm{\pi }}{\mu }_{0}^{2}{n}_{{THz}}^{2}{n}_{p}^{2}}\exp \left(-\frac{{\tau }^{2}{\Omega }^{2}}{8\mathrm{ln}\left(2\right)}\right){{\rm{sinc}}}^{2}\left(\frac{\Delta k\left(\Omega \right)L}{2}\right)$$

expression gives a good approximation of the THz energy spectrum^43^, where $L$ is the crystal length, $\Omega$ is the angular frequency, ${d}_{{eff}}$ is the nonlinear optical coefficient, ${I}_{0}$ is the peak intensity of the pump, *τ* is the FWHM of the pump pulse duration, ${\mu }_{0}$ is the vacuum permeability, *n*_*THZ*_ and *n*_*p*_ are the phase refractive index for the THz and pump pulse, respectively. The phase-mismatch is

2 $$\Delta k\left(\Omega \right)\cdot L\approx \frac{\Omega }{c}\left({n}_{{THz}}\left(\Omega \right)-{n}_{p}^{{gr}}\left({\omega }_{0}\right)\right)\cdot L$$

where *c* is the speed of the light and ${n}_{p}^{{gr}}\left({\omega }_{0}\right)$ is the group refractive index of the pump pulse. Hence the condition for phase matching, $\Delta k=0$, is only fulfilled if the THz-related phase refractive index and the pump-related group index are equal. Or, equivalently, if the phase velocity of THz and the group velocity of the pump is equal:

3 $${v}_{{THz}}={v}_{p}^{{gr}}$$

In case of velocity matching Eq. 1 has a maximum at $\Omega =\frac{2\sqrt{2\mathrm{ln}\left(2\right)}}{\tau }$. Hence, in case of a lossless medium the ${\nu }_{0}$ peak frequency of the generated THz radiation is:

4 $${\nu }_{0}=\frac{\sqrt{2\mathrm{ln}\left(2\right)}}{\pi }\,\cdot\, \frac{1}{\tau }$$

thus, the THz frequency can be tuned by the pulse duration of the pump pulse. The spectral bandwidth is 1.16 times the peak frequency. Accordingly, in a lossless medium, for 100 fs pump pulse duration, the peak THz frequency and bandwidth are ${\nu }_{0}=3.75$ THz and $\Delta \nu =4.33$ THz, respectively.

**Fig. 2:**
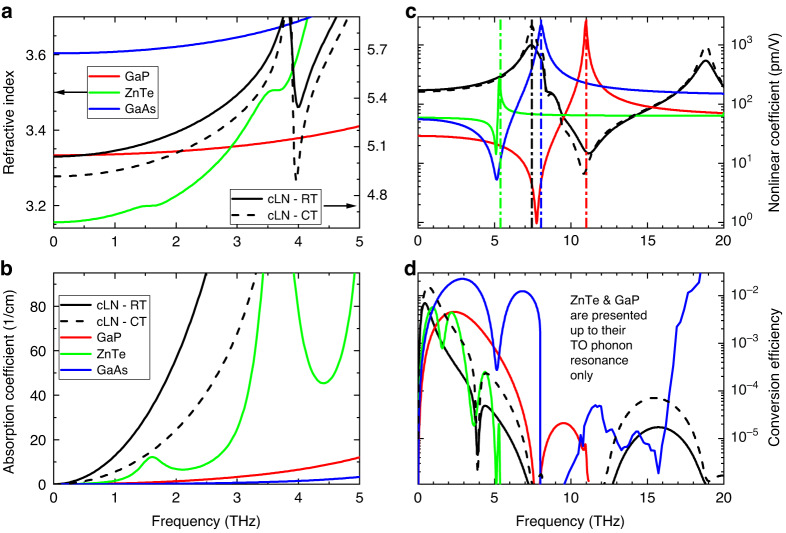
Most important parameters of GaP, ZnTe, GaAs, and LN in the THz range. Refractive index (**a**) and absorption (**b**) between 0 and 5 THz.The refractive indices increase with THz frequency. LN has a significantly larger refractive index (right scale). The effect of cryo cooling can be observed (RT – room temperature, CT – cryocooled temperature). LN owns the highest absorption, even when it is cooled down. The lowest absorption in the entire range among all the NM-s belongs to GaAs. Dispersion of the nonlinear coefficient (**c**) and conversion efficiency based on (5) between 0 and 20 THz (**d**). The vertical lines in (**c**) present the lowest transverse-optical (TO) phonon resonances. The highest and the smallest nonlinear coefficients at low frequencies correspond to LN and GaP, respectively. According to (**d**), below 1.5 THz LN proves to be the most favorable, while between 1.5 and 4.5 THz, GaAs exhibit superior characteristics. 1 *μ*m pumping wavelength was assumed for the determination of the nonlinear coefficients

In reality, the peak frequency and bandwidth are strongly affected by the dispersion and absorption in the THz range of the applied nonlinear crystal. The stronger the THz dispersion and the longer the crystal, the narrower the spectral bandwidth of the generated THz radiation. Figure 2a presents the THz refractive indices of the most commonly used crystals (LN – right scale, ZnTe, GaP, GaAs – left scale) for THz generation. Generally, approaching zero frequency the refractive index become frequency-independent, while at higher frequencies, close to the lowest transverse optical phonon frequency of the given material, the refractive index increases rapidly. Figure 2b shows the THz absorption of the same materials. The absorption increases monotonically with the frequency, leading to the suppression of higher-frequency components. One can observe that semiconductors have significantly lower absorption than LN at preferably large spectral range. The absorption of LN can be strongly reduced by cooling it to 100 K^44^.

Taking into account the THz absorption, the conversion efficiency of velocity matched THz generation at $\Omega$ angular frequency can be written as^34^

5 $$\eta =\frac{2{\Omega }^{2}{d}_{{eff}}^{2}{L}^{2}I}{{\varepsilon }_{0}{n}_{p}^{2}{n}_{{THz}}{c}^{3}}\exp \left(-{\alpha }_{{THz}}L\right)\frac{{\sinh }^{2}\left({\alpha }_{{THz}}L/2\right)}{{\left({\alpha }_{{THz}}L/2\right)}^{2}}$$

where *I* is the pump intensity, and ${\alpha }_{{THz}}$ is the THz absorption. The conversion efficiency depends squarely on the effective nonlinear coefficient, ${d}_{{eff}}$, which is not equal to the ${d}_{{eff}}$ value valid for the optical frequency conversion processes (i.e. SHG), rather can be determined by^34^

6 $${d}_{{eff}}=-\frac{{n}_{p}^{4}{r}_{{eff}}}{4}$$

where ${r}_{{eff}}$ is the clamped electro-optic coefficient of the material. Figure 2c shows the frequency dependence of the nonlinear coefficient for the four discussed materials on a broad frequency range. A rapid change of the value of ${d}_{{eff}}$ can be observed for all the materials around the transverse-optical (TO) phonon resonances indicated by vertical lines. The order of the location of the minimum, and maximum close to the TO phonon resonance is the opposite for LN and semiconductors. The reason for this is that the signs of the nonlinearities of electrons and phonons are different in semiconductors, while they are identical in LN. Also, for this reason the nonlinear coefficient, valid for OR, is smaller in semiconductors, while in LN is preferably (more than 5 times) larger than what is valid for optical frequency conversion. In the case of LN, approaching the phonon frequency on the low-frequency side, the enhancement of ${d}_{{eff}}$ can be observed. However, these large ${d}_{{eff}}$ values can not be utilized, due to the strong increase of the THz absorption on the same frequency range as can be seen in Fig. 2b.

Assuming $I=10$ ${\rm{GW}}/{{\rm{cm}}}^{2}$ pumping intensity for the presented materials, one can get the efficiencies in Fig. 2d, according to (5). Based on this, it can be stated that if the velocity matching is satisfied, for low frequencies ( < 1 THz) cryogenically cooled LN is the most suitable, while for higher frequencies (1.5–4.5 THz), GaAs is the best. For efficient THz generation, the fulfillment of (3) velocity matching condition is necessary. Since the refractive index of LN in the few THz regime is approximately twice as much as the visible and infrared group indices, despite its large nonlinear coefficient LN is unsuitable for collinearly THz generation. For ZnTe, GaP, and GaAs, there are pumping wavelengths (0.81 μm, 1.0 *μ*m, 1.36 *μ*m) available for collinear phase matching at 2 THz, however, at these wavelengths the pumping pulses with a few GW/cm^2^ intensities already generate a large number of free carriers owning to MPA. The generated free carriers inflict significant absorption at low THz frequencies^34,45–47^. For this, even the $I=10$ ${\rm{GW}}/{{\rm{cm}}}^{2}$ pumping intensity is too large to be used, thus the conversion efficiency of THz generation can not exceed the 0.01% level (see Fig. 1b, empty triangles).

The reason that the conversion efficiency could be larger by several orders of magnitude is the application of TPFP, which enables, on the one hand, the velocity matching in LN despite that the THz refractive index is significantly larger than the optical refractive index^34,35^, and on the other hand, in case of semiconductors, it also enables the velocity matching for pumping wavelengths long enough to suppress the low order MPA^31–34^.

## Tilted pulse front pumping technique for terahertz pulse generation in Lithium Niobate crystal

**Fig. 3:**
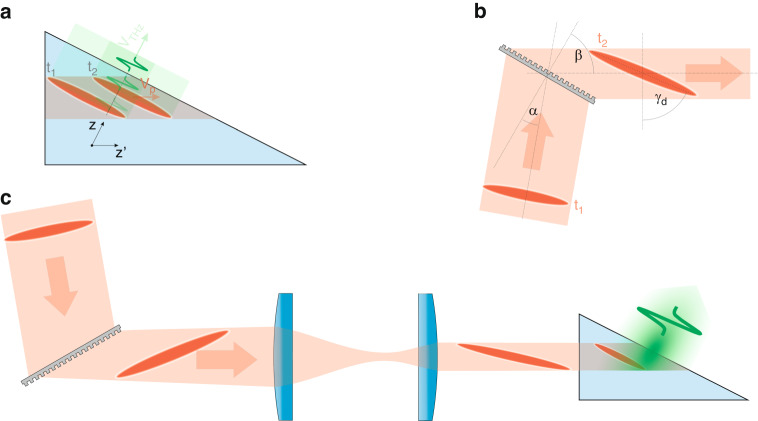
Conventional tilted pulse front setup. **a** Schematic figure of the propagation of the tilted pump pulse front along the z’ direction, and the propagation of the generated terahertz radiation along the z direction. Two snapshots at *t*_*1*_ and *t*_*2*_ moments are indicated together. **b** The schematic figure highlights the effect of the transmission grating, causing the initially normal pump pulse to become tilted in the setup. **c** Schematic figure of the entire conventional TPFP setup for THz generation, indicating four snapshots

The mechanism of the tilted pulse front setup is illustrated in Fig. 3a. The intensity front of the pump pulse is tilted by a $\gamma$ angle compared to the phase front, which is perpendicular to the propagation. The pump propagates to $z{\prime}$ direction, while the generated THz radiation propagates perpendicularly to the pulse front, to the direction of $z$. According to the figure, the pump pulse front and the phase front of the generated THz radiation stay on each other during their propagation (along different directions), and so the THz radiation can continuously get stronger if the

7 $${v}_{{THz}}={v}_{p}^{{gr}}\cos \left(\gamma \right)$$

relation is satisfied^35^. In the case of pulse front tilt, Eq. (7) has to be fulfilled instead of the velocity matching condition (3). This can be achieved with suitable $\gamma$ in all the cases where ${\nu }_{{THz}}\le {\nu }_{p}^{{gr}}$. This necessary tilting angle for congruent LN at 1030 nm pumping and velocity matching at 0.5 THz, depending on the crystal temperature, is between 62.0-63.4°^48^.

Pulse front tilt can be introduced by a dispersive optical element^49,50^, like by a prism or a grating. However, in the case of LN, the necessary large tilt angle is not feasible by a prism, thus the pulse front tilt is set by reflection or transmission diffraction grating.

The pulse front tilt introduced by diffraction off the grating is

8 $$\tan ({\gamma }_{d})=\frac{\sin \left(\alpha \right)+\sin \left(\beta \right)}{\cos \left(\beta \right)}$$

according to Fig. 3b, where $\alpha$ is the angle of incidence and $\beta$ is the angle of diffraction.

Diffraction on the grating not only produces pulse front tilt, but angular dispersion as well^49–51^, thus temporal and spatial chirp is created during the propagation^35,51–53^, the tilting of the pulse front decreases, and the pulse duration expands^51^. To avoid these distortions and to have proper control of the pulse front tilt, the conventional tilted pulse front scheme includes an imaging part (lens^35^, or telescope^36^), according to Fig. 3c. In the case of flawless imaging the beam is free from spatial chirp in the imaging plane and the pulse duration is equal to the value before the diffraction on the grating. However, the tangent of the pulse front tilt decreases with the amount of the magnification *(M)* of the imaging. (Usually, cases with $M < 1$ are applied, this is why the tilting angle increases.) As the pump pulse enters into the NM the tangent of the tilting angle decreases by the *n*_*g*_ group refractive index of the NM, thus the tilting angle inside the NM is

9 $${\gamma }_{M}={\rm{atan}}\left(\frac{1}{M\cdot {n}_{G}}\tan {\gamma }_{d}\right)$$

The tilting angle should fulfill the velocity matching condition (7). The tilting in air $\left({\gamma }_{A}\right)$ before the NM should be larger than this according to: $\tan \left({\gamma }_{A}\right)={n}_{g}\cdot \tan \left({\gamma }_{M}\right)$. This *γ*_*A*_ in the case of LN is between 76.4° and 77.2° depending on the temperature.

According to the

10 $$\sin \left(\alpha \right)+\sin \left(\beta \right)=\frac{\lambda }{p}$$

grating equation, where $\lambda$ is the wavelength of the incident light and *p* is the grating period, Eq. (8) can be written as

11 $$\tan ({{\rm{\gamma }}}_{d})=\frac{\lambda }{p\sqrt{1-{\left(\frac{\lambda }{p}-\sin \left(\alpha \right)\right)}^{2}}}$$

At a given grating with a *p* grating period, the required pulse front tilt angle ${\gamma }_{M}$ can be produced by an incidence angle of

12 $$\alpha ={\rm{asin}}\left(\frac{\lambda }{p}\pm \sqrt{1-\frac{{\lambda }^{2}}{{p}^{2}{n}_{g}^{2}{M}^{2}{\tan }^{2}\left({\gamma }_{M}\right)}}\right)$$

Since the diffraction efficiencies of the gratings are maximal at Littrow configuration $\left(\alpha =\beta \right)$, it is convenient to implement a setup close to this.

As evident from Fig. 1, the introduction of the TPFP technique has led to a substantial increase of several orders of magnitude in both the generated THz pulse energies and the conversion efficiencies within a few years.

Sadly, the several 10% efficiencies of the nonlinear frequency conversion processes, usual in the optical range, are unachievable for THz generation by OR. The main reason for this is that the conversion efficiency is proportional to the square of the generated frequency (see Eq. 5), and the latter is smaller by a few hundred times for OR than for SHG. In the case of LN, the available conversion efficiency is strongly limited by the significant absorption in the THz range (Fig. 2b), too. However, its nonlinear optical constant, *d*, is five times higher in the THz than in the optical regime (Fig. 2c). For considerably high conversion efficiency a high pumping intensity is necessary (in the case of LN ~ 100 GW/cm^2^ level). This pumping intensity is not limited primarily by the damage threshold (which, in the case of LN can be higher than 1 TW/cm^2^ for fs pulse duration), rather the temporal- and spectral distortion of the pump owning to the nonlinear refractive index, which has drastic consequences on the process of THz generation^54–56^. According to numerical calculations, the maximum obtainable conversion efficiency is around 1–2%^54–56^.

It should be noted that since the average photon energy of a pumping photon of, for example, 1.03 μm is more than 500 times higher than a photon of 0.5 THz, a 1% energy conversion efficiency equivalent to a photon conversion efficiency of 500%. Throughout THz generation, the intensity of the higher frequency components of the pump is decreasing, while the intensity of the lower frequency components is increasing, thus the pump spectrum shifts to the red during OR^57^. Since only a part of the THz radiation generated by OR leaves the LN crystal, as a consequence of THz absorption and Fresnel reflection at the output surface, the amount of the observable relative redshift of the pump can be typically 2–10 times of the one corresponding to the conversion efficiency.

Of course, the remarks on the conversion efficiency in the last two paragraphs are not true to the TPFP setup only, but to every OR process. There is, however, one more detrimental property of the TPFP setup on the available efficiency: Together with the pulse front tilt there is always present angular dispersion in the beam^49–51^ which leads to group delay dispersion (GDD)^50,51,58^. This GDD coming from TPFP in the case of LN with a pulse front tilt of γ = 63° is more than one order of magnitude bigger than the GDD coming from the material dispersion of LN! For the strong GDD, the ultrashort pump pulse duration will expand quickly during the propagation, thus THz generation is only effective in a short crystal length (the effective generation length is short^59^). This efficiency-reducing effect can be decreased by using longer FT-limited pump pulses, according to theoretical considerations^59^, and experiments^60–62^. Theoretically, the generated THz pulse energy can be increased even at a given conversion efficiency just by increasing simultaneously the pumping energy and spot size. The latter one is required since the pumping intensity can not be increased to any extent, since: (i) the damage threshold of the nonlinear material, (ii) the intensity dependent strength of the nonlinear refractive index, and (iii) the MPA. However, one should avoid simultaneously increasing the pumping energy and spot size beyond a certain level. Both the experiments^63^ and the numerical calculations^54–56^ show that by increasing the pump spot size, the conversion efficiency increases first (since the propagation direction of the pump and the THz is different, and the effect of the corresponding walk-off is smaller for larger pumping spot size), but after a certain size it significantly drops. There are two primary reasons for the decrease in conversion efficiency when using excessively large spot sizes: (i) imaging error (in the plane of dispersion) and (ii) prism shape of the crystal. Both effects are amplified as the tilting angle required for velocity matching increases. In the case of LN, where $\gamma \approx 63^\circ$, these limitations become more pronounced compared to semiconductors, where $\gamma \approx 30^\circ$ (Fig. 7).

As it was mentioned above, the diffraction grating illuminated by the pumping beam has to be imaged into the NM for the THz pulse to be generated by a pumping beam that has the same pulse duration (usually Fourier limited) as it had before the diffraction, and has no spatial chirp^22,35^. Imaging both with a single lens or with a confocal telescope (relay-imaging) can be used^21,22,28,31,32,34–36,54,55,64,65^. In case of low pumping energy ( < 1 mJ) spherical lens(es)^22,28,31,32,35,36,66^, in case of high pumping energy ( > 100 mJ) cylindrical lens(es) should be applied^40^ for providing the appropriate pumping intensities. In order to obtain maximum pump-to-THz conversion and optimal THz beam characteristics in a conventional TPFP setup the following conditions have to be fulfilled^64^: (i) Velocity matching of pump and THz requires a certain tilt angle γ of the pump pulse front inside the crystal given by Eq. (7). (ii) The pump pulse front has to be plane in the crystal. (iii) The image of the grating should be parallel with the pulse front. This alignment ensures that the pump pulse duration remains equal to the transform-limited value along the entire tilted pulse front. According to geometrical optical considerations, in paraxial approximation, all the above three conditions are fulfilled simultaneously^59,64^ if the sine of the incidence angle on the grating is equal to

13 $$\sin \left({\theta }_{i}\right)=\frac{{\lambda }_{0}}{p}\left(1-\frac{a}{n\cdot {n}_{g}}\right)$$

where ${\lambda }_{0}$ is the average wavelength of the pump, *p* is the period of the grating, *n*, and *n*_*g*_ is the (phase) index and group index of the NM,

14 $$a=\frac{{n}^{2}{n}_{g}p}{2{\lambda }_{0}}\sqrt{\frac{{\lambda }_{0}^{2}}{{n}_{g}^{2}{p}^{2}{\tan }^{4}\gamma }+\frac{4}{{n}^{2}}}-\frac{{n}^{2}}{2{\tan }^{2}\gamma }$$

and the magnification of the imaging system (the single lens or the two-lens telescope) is

15 $$M=\frac{1}{\sqrt{a}}$$

For an Yb-laser pumping (*λ* = 1.03 μm) these eqations are satisfied with good accuracy using for example an optical grating with 1/*p* = 1400 mm^-1^ groove density at 37.6^o^ incidence angle and a telescope with *M* = 2/3 (details are given in Ref. ^64^).

**Fig. 4:**
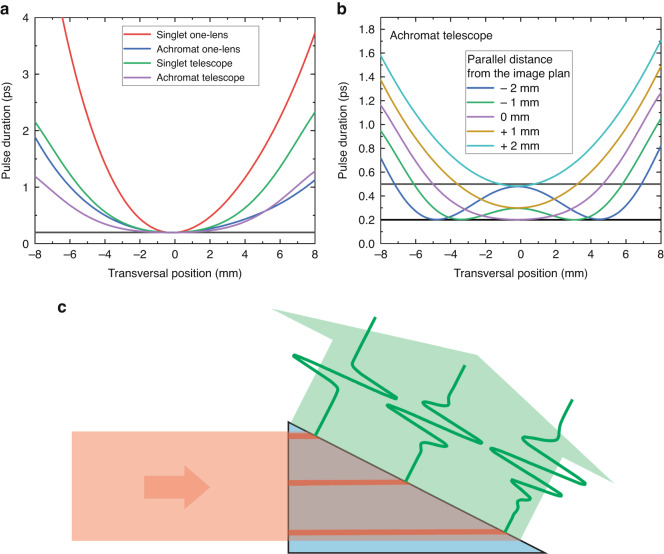
Distortions occurring in conventional setup at large cross-section. **a** Local pump pulse length along the paraxial image plane for 1.03 μm pump wavelength and a Fourier-limited pulse duration of 200 fs. The red line represents the singlet one-lens, the blue line represents the achromat one-lens, the green line represents the telescope consisting of two singlet lenses, and the purple line represents the telescope consisting of two achromat lenses. **b** Local pump pulse length at planes parallel to the paraxial image plane situated a few millimeters away from it, specifically in the case of an optimized achromat telescope. **c** The schematic picture illustrates the prism-shaped crystal, where the pump beam propagates different path lengths across various transversal positions. As a result, the generated THz pulse shape exhibits variations and inhomogeneity along the transversal position of the THz beam. **a**, **b** Reprint with permisison from [64]. *Copyright © 2016, Springer Science Business Media New York*

However, in non-paraxial cases (finite spot size), based on ray-tracing calculation, in the presence of angular dispersion, the imaging errors cause significant distortions, for example: curved images and curved pulse fronts^59,64^. The strongest limitation on the maximum spot size of the pump comes from the difference between the curvature of the image and the curvature of the pulse front. For this reason, as depicted in Fig. 4a, the pulse duration varies with the transverse position within the cross-section of the beam in the plane of the angular dispersion in the paraxial image plane, and it can be significantly larger than its Fourier-limited value^64^. The shorter the Fourier-limited pulse duration, the larger this pulse lengthening.

From the modeling of four different imaging systems (singlet or achromatic single-lens, and telescope consisting of singlet or achromatic lenses, respectively), the achromatic lenses are more advantageous (see Fig. 4a) and using a telescope will decrease the pulse lengthening at the edges of the beam compared to using a single lens. Moreover, the telescope causes less curved pulse front tilt, therefore the radius of the phase front of the exiting THz beam from LN reaches 1 m^64^, thus the divergence of the beam will be small. In contrast, for a single lens, the radius of curvature is 6 cm only. So, regarding a single lens, the THz beam owns a large divergence and a significant astigmatism, since in the case of a spherical lens in the plane perpendicular to the angular dispersion, a much longer radius of curvature is expected, and in the case of a cylindrical lens, the radius of curvature in this plane is even infinitely long. These problems can be reduced by using a lens with a longer focal length^59^, but the best is using telescope for imaging^64^. It should be noted, that the generating setup with which the highest THz pulse energy was delivered (1.4 mJ) so far, consisted of a telescope^40^.

According to Fig. 4b, the relationship between the local pulse duration and the transverse position varies with the longitudinal position in the vicinity of the imaging plane. The reason for the behaviors presented in Fig. 4a and b is the presence of the angular dispersion in the pulse front tilted beam^50,51,58^, which causes group velocity dispersion (GDD)^50^, and the curved image surface. After diffracting off the grating, the duration of the pump pulse undergoes rapid changes throughout its propagation.

As depicted in Fig. 4c, the prism shape of the LN crystal causes the pump pulse to travel varying distances across its cross-section, even without considering imaging errors. It is shown that the path length of the pump beam is considerably shorter on the same side as the apex of the prism, compared to the opposite side. Moreover, the path lengths of the THz radiation generated at various positions within the cross-section are significantly influenced by the transversal position. Consequently, the resulting THz pulse exhibits non-uniformity both spectrally and in the temporal evolution of the electric field^55,67,68^. This inherent inhomogeneity poses limitations on achieving strong focus as well.

Despite the substantial increase in the generated THz pulse energy achieved by employing conventional TPFP in an LN nonlinear medium (as illustrated in Fig. 1a), it is clear that addressing the aforementioned challenges is crucial for enabling the generation of higher energy THz pulses with significantly higher peak electric fields. Consequently, there is a pressing need to eliminate or at least mitigate the issues mentioned earlier. Overcoming these limitations will pave the way for the production of higher energy THz pulses with enhanced peak electric field characteristics.

## New-generation terahertz sources

**Fig. 5:**
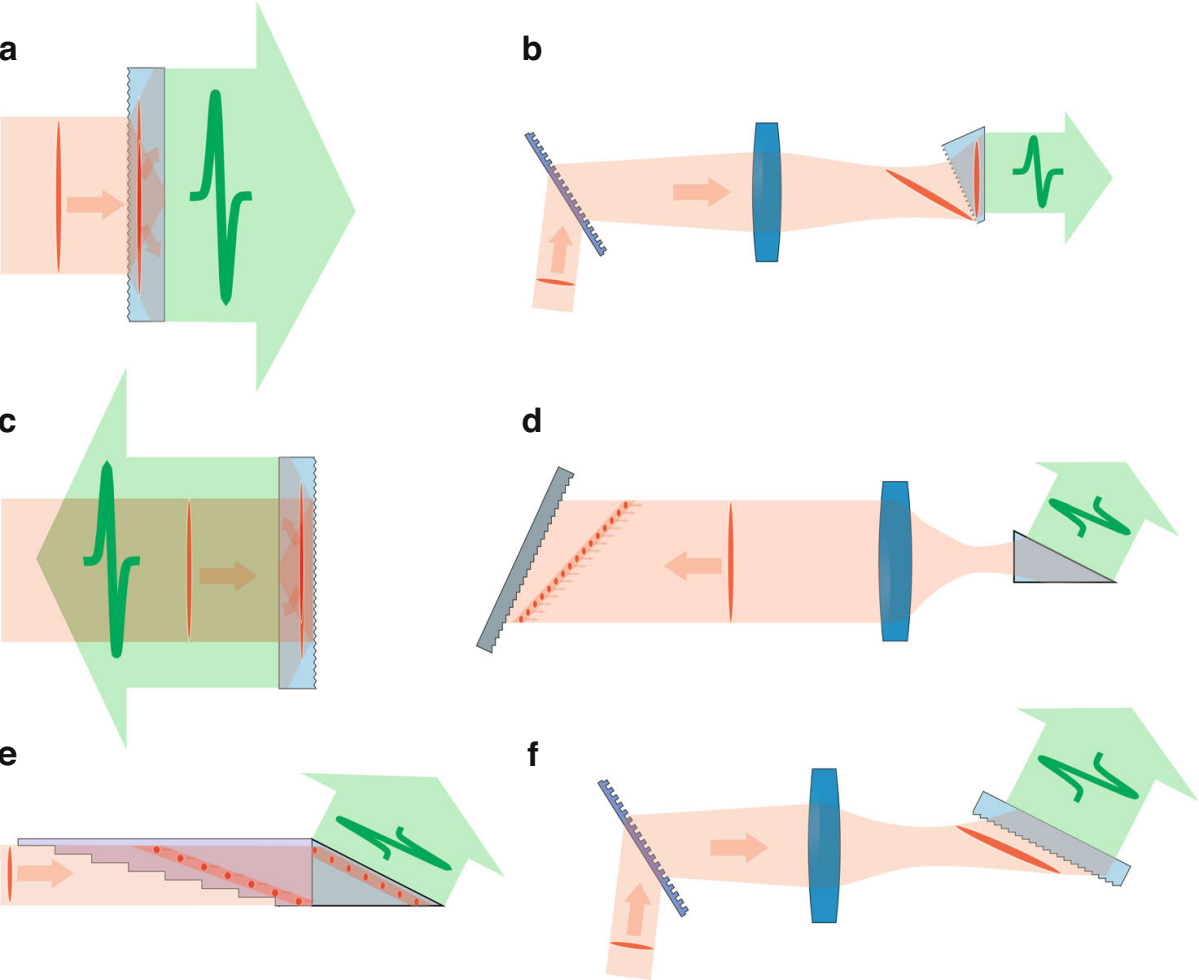
New-generation THz sources. **a** Contact grating scheme, where the diffraction grating produces the pulse front tilting is created on the surface of the crystal. Inside the NM the pump beams propagate along changed directions, but the intensity front remains parallel to the crystal surfaces. **b** Hybrid contact grating scheme, where the ${\gamma }_{M}$ pulse front tilt is created by the conventional external grating and the CG on the crystal surface together. **c** Reflection nonlinear slab (RNLS) scheme, where the pulse front tilt is created on the back microstructured surface of the nonlinear crystal. Velocity matched THz generation is realized during the backward propagation of the two diffracted beams on the back surface of the crystal. **d** Conventional TPFP scheme where the external optical grating is replaced by an echelon grating. The tilted pulse front is segmented. **e** Multistep phase mask scheme, where the segmented pulse front tilt is produced by a periodic dielectric structure connected to the nonlinear crystal. **f** Nonlinear echelon slab (NLES) scheme utilizes an external grating to create a tilted pulse front with a tilt angle of γ_M_ in the air, just in front of the nonlinear crystal. Additionally, a microstructured echelon-step crystal surface is employed to maintain the average tilt angle during the entrance into the NM

Pálfalvi et al. proposed a new setup idea, free from the aforementioned obstacles related to the increase of the pump beam size, the so-called contact grating (CG) THz source in 2008^65^. Here, the pulse front tilt is introduced not by the diffraction on a separate optical grating, but on the CG structure realized on the entry surface of the NM crystal as can be seen in Fig. 5a. The incoming pump beam hits the CG perpendicularly, then the two beams created by the diffraction on the CG propagate with ${\pm \gamma }_{M}$ angle to the original propagation direction creating the right pulse front tilt to generate THz radiation. There is no need for imaging and plan-parallel crystals can be used. Nevertheless, the implementation of this elegant and ideal setup is technically difficult in LN for the enormous pulse front tilt angle and the correspondingly high diffraction angle. As it was shown by Nagashima et al. ^69^, if the pump beam arrives at the rectangular grating profile on LN from air, then the diffraction efficiency is only 40%. This can be increased up to 90% if it comes from bulk quartz^69^ or velocity-matching liquid^70^. The coupling-in efficiency into the crystal can be improved by evaporating a Fabry-Perot structure between the crystal and the grating^71^. With this structure, a diffraction efficiency of 71% was achieved (where the grating period was 420 nm), although the THz conversion efficiency (partially because of the high THz coupling-out losses and the disadvantageously long 1 ps pump pulse duration) was only 0.015%. With the setup shown in Fig. 5a, but using semiconductors instead of LN, conversion efficiencies above 1% are expected^33,72,73^ for the significantly smaller pulse front tilt angle (see details in the following chapter).

For easier manufacturing the hybrid setup as depicted in Fig. 5b was proposed^74^. Here the pulse front tilt is introduced in two steps, by a separate transmission grating and by the contact grating itself. This imposes a less challenging material processing^74^. In the case of LN the grating period can be 620 nm instead of 420 nm^74^. This setup also contains imaging, however, the imaging error going to be more than three times smaller compared to the conventional TPFP setup since only half of the pulse front tilt is created by the separate grating. In this system the NM is also prism-shaped, however, its angle is advantageously less than half compared to the prism in the conventional setup (LN: 30.2° instead of 63.2°) leading to a much more homogeneous THz beam.

In Fig. 5c, the grating structure is not on the entry surface of the plan-parallel NM, but on its back surface. This one is called a reflective nonlinear slab, RNLS^75,76^. The operating principle is the following: The pump beam enters perpendicularly into the crystal and goes all the way to the reflective diffraction grating. There is no velocity matching up to this point, thus THz generation does not occur either. However, after the diffraction in the $\pm {1}^{{st}}$ (or higher) orders with an angle of ${\gamma }_{M}$ as the pump beam propagates backward in the NM, the velocity matching in (7) going to be satisfied and the pumping intensity front is parallel with the front and back surfaces of the crystal, thus the generated THz pulse leaves the crystal perpendicularly through the front surface. The RNLS, similarly to the CG (Fig. 5a), can be increased in its cross-section enabling the upscaling of the energy of the THz pulses by the beam diameter and pump energy. Moreover, the propagation of the pump pulse inside the NM is identical along the cross section of the crystal, thus the beam quality of the generated THz radiation is excellent, and the time evolution of the electric field is uniform along the cross-section. In a newly proposed version of RNLS, both the front and back surfaces are plane and the reflection and diffraction of the pump beam does not occur inside the NM, but on the grating structure right behind the crystal, which is realized on a material that is easier to process than LN^76^. In this case, there is a medium with a higher refractive index than LN, which is located between the NM and the grating and provides optical contact. This can be i.e. selenium or As_2_Se_3_. According to the numerical calculations the optimal crystal length is only a few mm^54,56^.

As it was mentioned in the previous chapter, in the conventional TPFP setup, in relation to the angular dispersion, the pulse duration changes with the propagation of the pump pulse. To avoid this Ofori-Okai et al. proposed a setup^77^, where instead of the optical grating of the conventional setup a reflective stair-step echelon introduces the pulse front tilt (Fig. 5d). Along the propagation direction, the spatial distance between the beamlets created by the reflection on the stair steps of $W$ width and $H$ height is $2H$. For this, the angle of the tilted pulse front is

16 $$\gamma ={atan}\left(2H/W\right)$$

Similarly to the conventional TPFP setup, the tangent of this varies according to what was mentioned before Eq. (9) for the magnification of the imaging and due to the entry of the pump pulse into the NM, thus the introduced pulse front tilt inside the NM is:

17 $${\gamma }_{M}={atan}\left(\frac{2H}{{MW}{n}_{g}}\right)$$

It should be noted that this pulse front tilt angle applies to the average pulse front tilt, the individual beamlets are not tilted, and the velocity matching is not fulfilled for them. Thus, the *w* size of the beamlets cannot exceed^78^

18 $$w=\frac{{2\lambda }_{{THz}}}{\pi {n}_{{THz}}\sin \left({\gamma }_{M}\right)}$$

where $w=W\times M$. From the other side, the beamlets cannot have any small size either, since as their initial size decreases, the divergence of the beamlets increases and after a few mm of propagation $\left({z}^{{\prime} }\right)$ the size of the beamlets will be so large that the $w$ averaged over the $0-z{\prime}$ traveling range will be larger than in the case of a larger initial $w$. The efficiency of this setup was experimentally demonstrated by ultrashort pulses^77^. With 1 mJ pulse energy, 39 fs FT limited pump pulses and a reflective stair-step echelon with $W=150$ $\mu m$ step height and applying $M=0.2$ magnification, they could achieve 0.33% conversion efficiency. Using a similar setup with 280 fs pump pulse duration, Guilamand et al. accomplished 1.3% efficiency proving that the segmented pulse front tilt can be efficient for longer than 100 fs pulse duration as well^79^.

Just like in the previous case, the aim of the following setup is also to avoid the pulse duration change coming from the angular dispersion, but here the pulse front tilt is introduced by a segmented phase mask, so called multistep phase mask (MSPM), placed on the NM^80^ (Fig. 5e). The setup is obviously free from imaging errors, however according to numerical calculations^56^ the achievable efficiency may be slightly smaller compared to the previous case, since the distortions of the pump beam are caused by diffraction as it propagates through the phase mask.

Noteworthy, is that both the last two setups have prism-shaped NM-s which definitely limits the upscaling of the size and THz energy.

The THz source proposed by Pálfalvi et al. enables the use of segmented pulse front tilt and plane parallel NM^81^. This hybrid-type setup is shown in Fig. 5f. The operating principle is as follows: The transmission grating and the imaging introduce that ${\gamma }_{A}$ pulse front tilt directly before the entry surface of the NM, which is needed inside the NM to the velocity matching according to (7) (in case of conventional setup, the required angle is greater, see below (9)). A step structure is formed on the entrance surface of the crystal (nonlinear echelon slab, NLES) with step height (H) and width (W) to satisfy:

19 $${\gamma }_{A}={atan}\left(\frac{H}{W}\right)$$

Thus, while the tilted pumping pulse enters through the steps, it is decomposing to beamlets with tilt angles of $\tan \left({\gamma }_{M}\right)=\tan \left({\gamma }_{A}\right)/{n}_{g}$ which is smaller than the average pulse front tilt *γ*_*A*_, that is unchanged. Hence the NM has plane parallel sides and every beamlet propagates identically (apart from imaging errors), the quality of the generated THz beam going to be excellent for even big pumping diameters as well^56^. The first implementation of a small-sized hybrid THz source setup containing NLES demonstrated its functionality^82^. Although the achieved efficiency was only 0.05%, but a tenfold increase is expected if the pump beam size and energy are increased and the NLES is cryocooled. Difficult to estimate, but further significant increase in the efficiency is likely from better-quality manufacturing of the NLES.

According to additional suggestion^67^, the imaging can be omitted by using wedged shape NLES with 8-10°. This angle can be further decreased if the transmission grating is used not in exactly Littrow configuration^83^. With the first implementation of the wedged shaped NLES 40 $\mu$J THz energy and 0.1% efficiency were demonstrated by 500 fs and 200 fs pump pulse durations^84^. This efficiency can be increased eightfold by cryocooling the NLES, using sLN as a crystal material, and better-quality manufacturing of the NM. According to the investigations, the wedged shaped NLES provides excellent beam quality^84^.

In the case of plane parallel NLES, the imaging can also be omitted by using volume holographic gratings^85^, hence they provide more than 90% diffraction efficiency even if they are not in Littrow configuration^86,87^.

A comprehensive comparison of the different TPFP schemes can be found in Table 1.

**Table 1:** Overview of the properties of the tilted pulse front setups

The color map highlights advantageous (green) and disadvantageous (red) properties of the setups: conventional, prism shaped (conv.), contact grating (CG), hybrid (HYBR), reflection nonlinear slab (RNLS), stair-step-echelon based setup (SSE), multi-step phase-mask based setup (MSPM), and nonlinear echelon slab with (NLES) and without (IF NLES – imaging free NLES). Abbreviations are the followings: req. required, mod. moderate, diff. difficult, cont continuous, seg. segmented

## High-energy semiconductor-based terahertz sources

As it was mentioned earlier, for some semiconductors the velocity matching (3) can be satisfied at near-infrared wavelengths, however in these cases the free carriers generated through low-order MPA inflict THz absorption.

**Fig. 6:**
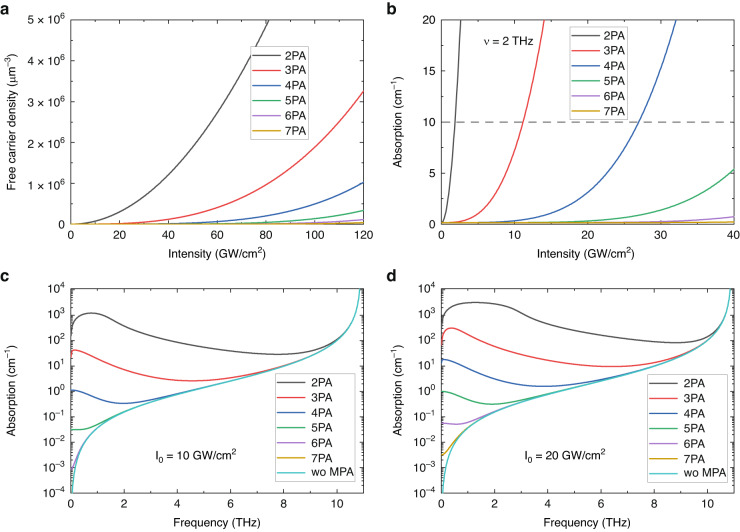
Comparison of the effects of different orders of MPA-s on absorption. **a** illustrates the approximation of the free carrier density dependence on pump intensity for different multiphoton absorption orders in GaP crystal. Using these values, (**b**) demonstrates the intensity-dependent free carrier absorption at a frequency of 2 THz. It is evident that for higher orders of multiphoton absorption, a higher intensity can be employed to achieve the same level of THz absorption. **c**, **d** Exhibit the THz spectrum of the phonon-polariton absorption alone (wo MPA) and the combined phonon-polariton and free carrier absorption, assuming pump intensities of 10 GW/cm² and 20 GW/cm², respectively. Notably, as the frequency increases, the difference between the absorption curves decreases. Moreover, at lower frequency ranges, if only higher-order multiphoton absorption occurs, the contribution of free carrier absorption becomes negligible

For a typical example, Fig. 6a shows the pumping intensity dependence of the created free carrier concentration for different levels of MPA and the resulting THz absorption at 2 THz (Fig. 6b) when GaP is pumped by 100 fs pulses. In Fig. 6c and d, the absorption spectrum caused by TO phonon-polariton alone (wo MPA, light blue) and by phonon-polaritons combined with different orders of MPA can be seen in case of pumping intensities of 10 (Fig. 6c) and 20 ${\rm{GW}}/{\rm{c}}{{\rm{m}}}^{2}$ (Fig. 6d). According to Fig. 6b, if GaP is pumped by a Ti:s laser with 800 nm wavelength, an absorption coefficient of 10 cm^-1^ is resulted even at 1.8 GW/cm^2^ pumping intensity due to the free carrier absorption coming from 2PA, which strictly limits the THz conversion efficiency. In case of pumping at 1.03 *μ*m by Yb lasers instead of Ti:s 3PA will be the lowest order MPA, and according to Fig. 6b, this enables to use of six-times higher pumping intensities, which can lead to six-times higher conversion efficiency. Moreover, according to Fig. 6b, by further increasing the pumping wavelength (>1.8 *μ*m), where 4PA dominates, the available pump intensity can be larger by an additional factor of two, and another twofold increase is expected in conversion efficiency. The longer wavelength provides significant (more than one order of magnitude by MPA orders) absorption decrease in a broad frequency range (see Fig. 6c and d) not only at 2 THz.

**Fig. 7:**
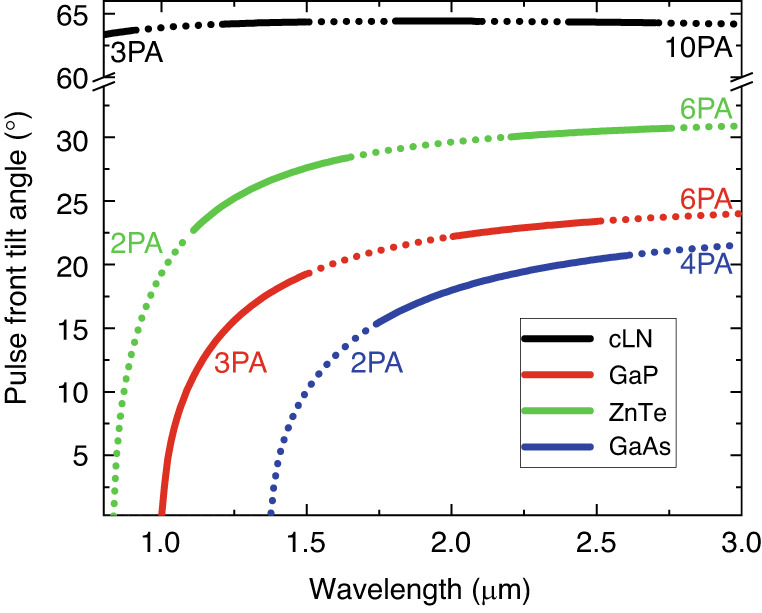
Pulse front tilt angle vs. pumping wavelength. The required pulse front tilt for velocity matching at 1 THz varies with the pump wavelengths for different semiconductors and LN. The dotted (solid) lines correspond to even-(odd-)order MPA-s

Suppression of the lower-order MPA by longer wavelength pumping has a huge advantage, but as mentioned, the velocity-matching condition (3) is generally not satisfied in this case. However, by using a tilted pulse front pumping with an appropriate γ angle, the velocity-matching condition (7) can be met. Figure 7 shows the dependence of the required pulse front tilt angle for velocity matching on the pumping wavelength for four different semiconductors and LN. The colors identify the semiconductors, the black curve corresponds to LN, and the solid and dotted sections indicate odd and even order MPA, respectively. As can be seen, for semiconductors the required pulse front tilt angle is advantageously only half of the 63° required for LN, even when using pumping with a long enough wavelength to allow only >6 order MPA.

**Fig. 8:**
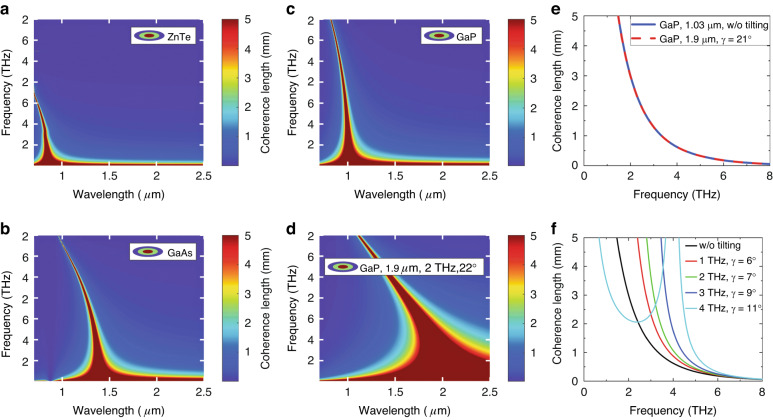
Coherence length of OR process in semiconductors. **a–d** Contour plot of coherence length versus the pump wavelength and THz frequency in ZnTe (**a**), in GaAs (**b**), and in GaP (**c**, **d**). In the case of (**a**–**c**) collinear pumping, while in the case of (**d**) TPFP with 21° tilt angle is supposed. This tilt angle resulted in a shift of the velocity-matching wavelength at 1 THz from 1.03 to 1.9 *μ*m. In the case of 1.9 μm pumping, only 4PA occurs, allowing for the utilization of higher pump intensities and leading to a significant enhancement in THz generation efficiency. **e**, **f** Coherence length versus THz frequency for OR in GaP. In the case of (**e**) collinear pumping with 1.03 mm (blue), and TPFP with $\gamma =21^\circ$, with 1.9 mm (red) is supposed. The two curves overlap, providing evidence that TPFP enables the utilization of longer wavelength pumping, which effectively suppresses low-order MPA, while simultaneously preserving the wide coherence length spectrum. **f** Illustration of the variation in the coherence length spectrum as the tilt angle changes for a constant pump wavelength. The legend indicates the velocity-matched frequency for different tilt angles

Since the refractive index also exhibits dispersion in the THz range (Fig. 2a), velocity matching is only fulfilled for a specific THz frequency. To characterize how the efficiency of THz generation decreases at frequencies different from this, it is useful to introduce the “coherence length” concept for the OR process, too. According to Eq. (1) (in the absence of THz absorption), if ${dk}\,\ne\, 0$, the efficiency of THz generation is maximal for

20 $$L=\frac{c}{2\cdot {\nu }_{{THz}}\left|{n}_{{THz}}-{n}_{p}^{{gr}}\right|}\equiv {l}_{c}$$

crystal length, which can be defined as the coherence length, ${l}_{c}$. Since the efficiency of THz generation is proportional to *L*^2^, a larger coherence length is very advantageous. Fig. 8a–d shows the contour plot of ${l}_{c}$ as a function of the pumping wavelength and THz frequency for different semiconductors. As can be seen in Fig. 8a, for example *l*_c_ > 5 mm can be achieved for 1 THz frequency in the case of ZnTe when the pumping wavelength is around 820 nm. This was exploited in Ti:s-pumped ZnTe THz sources for a decade. However, it can also be seen that *l*_c_ > 5 mm only applies for $\nu$ < 2 THz, while for GaAs and GaP, the *l*_c_ > 5 mm inequality is fulfilled for a wider THz frequency range (0–3 and 0–4 THz, respectively) for the appropriate pumping wavelength (1.35 and ≈1.0 µm, respectively) (see Fig. 8b and c). Accordingly, with these materials (as their THz absorption is also more favorable), wider bandwidth THz pulses or THz pulses tunable over a larger frequency range can be generated.

Comparing the three materials from the perspective of MPA, it can be concluded that 2PA occurs when pumping ZnTe at a wavelength of 0.82 μm or GaAs at a wavelength of 1.35 μm, while 3PA is the lowest-order MPA in GaP when pumped at approximately 1.0 μm. Recognizing this, ≈1.03 μm wavelength Yb-fiber lasers were used to pump GaP-based THz sources^27^. The importance of avoiding lower-order MPA was also demonstrated experimentally by OR with ZnTe and GaP using ≈1.03 μm laser pulses^28^. Although the non-linear optical coefficient of ZnTe is approximately three times larger than that of GaP, significantly higher efficiency was achieved using GaP, in which 3PA was the lowest-order MPA, compared to ZnTe, in which 2PA also occurred^35^. THz pulses were generated in GaAs by OR with 1.8 μm laser pulses with an efficiency of 0.05%^32^. In this case, as with pumping at ≈1.03 μm wavelength in ZnTe, velocity matching was ensured by using TPFP arrangement. By pumping ZnTe crystal at 1.7 μm (beyond the 3PA edge), it was possible to use a pump intensity as high as 15 GW/cm^2^, resulting in two orders of magnitude larger THz generation efficiency (0.7%) compared to pumping at 0.8 μm^31^. In this case, velocity matching was also ensured by using a TPFP arrangement.

The effect of velocity matching using TPFP on the pumping wavelength and THz frequency dependence of *l*_c_ is shown in Fig. 8c and d. In the case of Fig. 8c, there is no pulse front tilt, while in the case of Fig. 8d, a pulse front tilt of 21 degrees is assumed, which results in velocity matching at 1.9 μm pumping wavelength for 1 THz. Although the shape of the ${l}_{c}$ contour plot changed in Fig. 8d, the ${l}_{c}\left({\omega }_{{THz}}\right)$ function remained unchanged. This is shown in Fig. 8e, where the curves corresponding to the two cases overlap perfectly. In conclusion, by employing TPFP, it becomes possible to utilize longer pump wavelengths, thereby avoiding low-order MPA, while simultaneously maintaining the broad spectrum of the THz pulses generated with enhanced efficiency.

It is worth noting that it is generally accepted that velocity matching is fulfilled in the case of collinear configuration for Yb-pumped GaP. However, this is only approximately true, even for frequencies below 1 THz the phase matching precisely achieved only around 1.005 $\mu m$. Thus, in this case, the application of TPFP can also be useful. This is illustrated in Fig. 8f. It can be observed that without TPFP, assuming a 5 mm thick GaP, the coherence length is significantly greater than the crystal thickness for frequencies only < 1.5 THz. This limits the bandwidth of the generated THz pulses and their generation efficiency. In contrast, by applying a pulse front tilt of *γ*=9°, for example, the coherence length exceeds 5 mm across the entire 0.0–3.6 THz range, enabling high-efficiency THz generation over this wide range. In Ref. ^88^, a CG formed on a 2 mm thick GaP crystal was used with a pulse front tilt of *γ*=11°, resulting in an observed increase in both the efficiency and bandwidth of THz generation.

Certainly, the novel TPFP THz source configurations introduced in the preceding chapter can also be implemented with semiconductors, offering even easier feasibility due to the reduced tilt angle requirement^65^^,80–85^^,^^88,89^. For example, by implementing a contact grating arrangement with a ZnTe nonlinear crystal as shown in Fig. 5a, and using 1.7 μm pump pulses with a pumping intensity of 8 GW/cm², an outstanding 0.3% THz generation efficiency was achieved among semiconductor sources^33^. The crystal damage threshold is above 100 GW/cm², but increasing the pumping intensity above 8 GW/cm² resulted in decreased efficiency due to the absorption of free charge carriers generated by 4PA.

The utilization of longer wavelengths allows for higher pump intensities to be employed, resulting in enhanced THz generation efficiency, while simultaneously avoiding the occurrence of 4PA, 5PA, etc. Currently, the Ho laser operating around 2.0 *μ*m is the longest wavelength high-energy femtosecond laser available. Pump pulses with longer wavelengths than this can be generated using an optical parametric amplifier (OPA), but their efficiency decreases with wavelength. Model calculations that examine the joint efficiency of the THz and pump sources and take into account the effect of the nonlinear refractive index of NM show that it is not worthwhile to use OPA with wavelengths longer than 2.0 or 3.0 μm for GaP and GaAs, respectively^90^.

However, in recent times, several manuscripts have appeared that aim to generate CO_2_ laser pulses with the shortest possible duration. Today, even ps-length CO_2_ laser pulses are available^91^, which can be further compressed using external optical devices^92–95^ or other alternative methods^96^. For pump pulses around 10 *μ*m generated by CO_2_ laser, it is likely that only the damage threshold of semiconductor crystals and other nonlinear optical processes such as second harmonic generation during THz generation or pump self-phase-modulation will limit the pumping intensity. Calculations indicate that a conversion efficiency of over 1% can be achieved in GaAs^97^.

## Pulse front tilt in organic materials

**Fig. 9:**
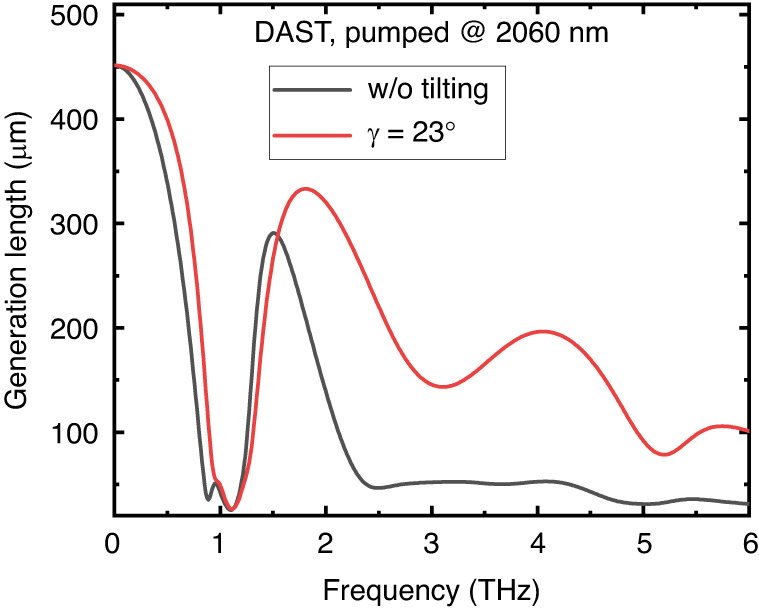
Effect of pulse front tilt on the generation length in an organic crystal. Generation length vs. THz frequency in DAST at 2.06 *μ*m pumping wavelength calculated by Eq. (21) in the case of collinear pumping (black line) and velocity matching by pulse front tilt of $\gamma =23^\circ$ (red line)

Until now, the utilization of TPFP in organic materials has not even been considered. This is probably due to two inherent properties of organic materials: (i) they have extraordinary large nonlinear coefficients, (ii) they can be grown only in the form of thin plates. Organic crystals are usually characterized by a THz frequency- and pump wavelength-dependent generation length which serves as figure of merit and is defined as follows^24^:

21 $${L}_{{gen}}\left(\omega ,\lambda ,z\right)=\sqrt{\frac{\left(\exp \left(-{\alpha }_{{THz}}\left(\omega \right)z\right)+\exp \left(-2{\alpha }_{{opt}}\left(\lambda \right)z\right)-2\exp \left(-\left[\frac{{\alpha }_{{THz}}\left(\omega \right)}{2}+{\alpha }_{{opt}}\left(\lambda \right)\right]z\right)\cos \left(\frac{\pi z}{{l}_{c}\left(\omega ,\lambda \right)}\right)\right)}{{\left(\frac{{\alpha }_{{THz}}\left(\omega \right)}{2}-{\alpha }_{{opt}}\left(\lambda \right)\right)}^{2}+\left(\frac{\pi }{{l}_{c}{\left(\omega ,\lambda \right)}^{2}}\right)}}$$

where ${\alpha }_{{THz}},{\alpha }_{{opt}}$ represent the absorptions in the THz and optical regime, and ${l}_{c}$ is the coherence length. To explore the potential impact of pulse front tilting, we should substitute ${n}_{g}/\cos (\gamma )$ instead of the group refractive index of the material ${n}_{p}^{{gr}}$ in the formula of coherence length (20). An insightful calculation involving a 0.5 mm thick DAST crystal pumped by 2.06 μm wavelength (the wavelength of a thulium-doped laser, or close to a holmium laser, which is much more widespread than a chrome forsterite laser with ~1.3 *μ*m, typically used for pumping organic crystals) demonstrates this phenomenon (Fig. 9). By using this long wavelength (similarly to ref. ^25^), 2PA can be avoided, however, without TPFP the velocity matching is lost. Figure 9. illustrates that pulse front tilting can lead to a significant increase in the generation length on a broad THz spectral range above 1.5 THz, thereby preventing the narrowing of the spectrum that would typically occur in the case of thicker crystals. The black curve corresponds to a collinearly pumped configuration. The first minima in the spectrum correspond to the high THz absorption of the crystal, while the subsequent minima above 2 THz are a result of velocity mismatch. However, by introducing an appropriate pulse front tilt (red curve), the generation length increases over the entire range above 1.5 THz. This effect stems from the ability of pulse front tilting to achieve an enlarged coherence length in this range. Importantly, the generation length has a direct connection to the conversion efficiency. Specifically, if the generation length expands by 3-4 times, the efficiency rises by the square of this increase. Leveraging this insight, it becomes possible to extend the lifespan of these vulnerable crystals by pumping them at substantially lower energies while still generating THz pulse energies with the same level in a more efficient way and the lower pumping enables to avoid cascading effects and the detrimental effect of the nonlinear refractive index.

## Detection of terahertz pulses

In principle, velocity matching is essential not only for THz pulse generation using OR, but also for THz pulse detection with EOS^98,99^. Nevertheless, the EOS technique using tilted pulse front probe pulses has not yet become widespread, probably because in this case the negative effect of the velocity mismatch on the detection bandwidth can be simply reduced by using a thinner EO crystal.

However, as recently demonstrated experimentally^88^, by ensuring velocity matching through the application of tilted pulse fronts in both THz generation and detection crystals, a combined OR and EOS-based time-domain terahertz spectroscopy (TDTS) system achieved a 400-fold increase in THz signal and a 35 dB larger dynamic range compared to using thin crystals. In this experiment a CG was used, similarly to (ref. ^33^), to achieve TPFP in the OR crystal, and the same technique was applied to the EOS crystal as well. This approach resulted in a compact setup. It is important to note that a 1.03 μm wavelength Yb laser was employed for pumping and detection, and GaP crystals for OR and EOS. This combination results in a relatively low level of velocity mismatch. If a 2 μm laser is used in GaP for OR (to achieve higher THz generation efficiency) and for EOS, a larger degree of velocity mismatch would occur in collinear arrangements, making the application of tilted pulse front velocity matching even more advantageous.

In addition to velocity matching, tilted pulse fronts have been also employed for single-shot THz pulse detection setups. In these configurations, the tilted pulse front was utilized to create a continuously varying time delay along the cross-section of the EOS probe beam, enabling single-shot measurements. Using optical grating to create a tilted probe pulse front an excellent measurement quality was demonstrated^100^.

## Summary

In this paper, the results achieved in the past two decades by using the TPFP technique for velocity-matching in OR-based THz sources were reviewed. The paper begins with a brief introduction to the conventional TPFP setup, followed by a discussion of the key design considerations. The advent of conventional TPFP THz sources marked a monumental breakthrough, propelling THz pulse energy from LN and semiconductors to unprecedented heights, and enabling widespread application of nonlinear THz measurements and material/process control experiments. Furthermore, it has unlocked a realm of possibilities for compact THz-driven particle acceleration, ushering in a new era of potential and advancement. However, several limitations associated with the conventional TPFP setup need to be addressed for the realization of this vision. In recent years, new-generation scalable TPFP THz source setups have been proposed and promising initial results have been demonstrated. Further technical development is required to improve the optical quality of the micro/nano-structured surfaces needed in these new-generation sources. The ongoing development is expected to yield highly efficient THz sources capable of routinely generating THz pulses with millijoules of energy. Furthermore, these sources not only facilitate the attainment of high energies but also enable the enhancement of beam quality and focusing, resulting in tens of megavolts per centimeter of electric field. This advancement significantly contributes to the field of extreme-field THz science and technology. THz driven acceleration, molecular orientation, material control, enhancement of high-harmonic-generation are just a few examples of areas which will strongly benefit from these developments.

It is also foreseen that applications requiring THz pulses with energy levels only in the μJ range, such as THz pump-probe measurements, will significantly benefit from the development of new-generation THz sources, such as semiconductor CG-s.

Additionally, exploring the application of TPFP with new nonlinear materials, such as organic crystals, holds promise for achieving further success. Moreover, it is essential to prioritize the development of the entire pump laser-THz source system, as it is vital for enhancing overall performance. Incorporating previously overlooked lasers, such as the efficient implementation of CO_2_ lasers for pumping, could notably enhance system efficiency, as demonstrated in this study.

In summary, TPFP THz sources have experienced significant success over the past two decades, and their future prospects are expected to be even more promising.

### Supplementary information

supplemental material for Tilted pulse front pumping techniques for efficient terahertz pulse generation
