# Supplementary material for: Tilted pulse front pumping techniques for efficient terahertz pulse generation
Source: Light Sci Appl. 2023 Oct 24;12:256. doi: 10.1038/s41377-023-01293-1 (PMC10593827; doi:10.1038/s41377-023-01293-1)
Supplement: Supplementary file 1 — supplemental material for Tilted pulse front pumping techniques for efficient terahertz pulse generation [file 41377_2023_1293_MOESM1_ESM.docx]

Supplementary Information for:

**Tilted pulse front pumping techniques for efficient terahertz pulse generation**

György Tóth^1,2,^^[[1]](#footnote-1)^† , Gyula Polónyi^2.3,^^[[2]](#footnote-2)^†, János Hebling^1,2,3^

*^1^University of Pécs, Pécs, 7624, Hungary*

*^2^Szentágothai Research Centre, Pécs, 7624, Hungary*

*^3^HUN-REN-PTE High-Field Terahertz Research Group, Pécs, 7624, Hungary*

1. **References for Figure 1.**

| **#** | **Material** | **Year** | **THz energy (**$\boldsymbol{\mu}$**J)** | **Efficiency (%)** | **Group** | **DOI** |
| --- | --- | --- | --- | --- | --- | --- |
| 1 | LN | 1971 | 0.1 | 5,00E-06 | Yang | 10.1063/1.1653935 |
| 2 | LN (TPFP) | 2003 | 9,80E-05 | 4,30E-03 | Stepanov | 10.1063/1.1617371 |
| 3 | Organic | 2003 | 7,00E+00 |  | Schneider | 10.1016/j.optcom.2003.07.013 |
| 4 | LN (TPFP) | 2004 | 4,00E-04 | 1,70E-02 | Hebling | 10.1007/s00340-004-1469-7 |
| 5 | ZnTe | 2005 | 5,40E-04 |  | Löffler | 10.1364/OPEX.13.005353 |
| 6 | LN (TPFP) | 2005 | 2,40E-01 | 5,00E-02 | Stepanov | 10.1364/OPEX.13.005762 |
| 7 | GaP | 2006 | 5,40E-14 | 7,64E-05 | Chang | 10.1364/OE.14.007909 |
| 8 | LN (TPFP) | 2007 | 0,1 | 2,50E-02 | Hoffmann | 10.1364/OE.15.011706 |
| 9 | LN (TPFP) | 2007 | 10 | 0,06 | Yeh | 10.1063/1.2734374 |
| 10 | GaP | 2007 |  | 1,62E-05 | Chang | 10.1364/OL.32.000433 |
| 11 | GaP (TPFP) | 2007 | 5,00E-03 | 1,25E-05 | Hoffmann | 10.1364/OE.15.011706 |
| 12 | ZnTe (TPFP) | 2007 | 1,10E-03 | 2,75E-06 | Hoffmann | 10.1364/OE.15.011706 |
| 13 | ZnTe | 2007 | 1,5 |  | Blanchard | 10.1364/OE.15.013212 |
| 14 | LN (TPFP) | 2011 | 3 | 0,1 | Hirori | 10.1063/1.3560062 |
| 15 | Organic | 2011 | 2,00E+01 | 2,2 | Hauri | 10.1063/1.3655331 |
| 16 | Organic | 2012 | 9 | 1,75 | Ruchert | 10.1364/OL.37.000899 |
| 17 | LN (TPFP) | 2012 | 125 | 0,26 | Fülöp | 10.1364/OL.37.000557 |
| 18 | Organic | 2013 | 0,1 | 4,00E-03 | Monoszlai | 10.1364/OL.38.005106 |
| 19 | Organic | 2013 | 15 | 0,8 | Ruchert | 10.1103/PhysRevLett.110.123902 |
| 20 | Organic | 2013 | 0,05 | 6,00E-03 | Monoszlai | 10.1364/OL.38.005106 |
| 21 | GaP | 2013 | 7,10E-06 | 1,47E-03 | Li | 10.1088/1612-2011/10/12/125404 |
| 22 | Organic | 2014 | 680 | 1,1 | Vicario | 10.1103/PhysRevLett.112.213901 |
| 23 | Organic | 2014 | 900 | 3,00 | Vicario | 10.1364/OL.39.006632 |
| 24 | LN (TPFP) | 2014 | 436 | 0,77 | Fülöp | 10.1364/OE.22.020155 |
| 25 | GaAs (TPFP) | 2014 | 17 | 0,05 | Blanchard | 10.1063/1.4904005 |
| 26 | Organic | 2015 | 700 |  | Vicario | 10.1088/2040-8978/17/9/094005 |
| 27 | Organic | 2015 | 62 | 2,1 | Vicario | 10.1364/OE.23.004573 |
| 28 | Organic | 2015 | 11 | 1,00 | Vicario | 10.1038/srep14394 |
| 29 | Organic | 2015 | - | 1,1 | Vicario | 10.1364/OE.23.004573 |
| 30 | Organic | 2015 | 109 | 2,86 | Shalaby | 10.1038/ncomms6976 |
| 31 | Organic | 2015 | 270 | 3,2 | Vicario | 10.1364/OE.23.004573 |
| 32 | Organic | 2015 | 76 | 2,14 | Shalaby | 10.1038/ncomms6976 |
| 33 | LN (TPFP) | 2016 | 2,1 | 0,21 | Ofori-Okai | 10.1364/OE.24.005057 |
| 34 | ZnTe (TPFP) | 2016 | 14 | 7,00E-01 | Polónyi | 10.1364/OE.24.023872 |
| 35 | ZnTe (TPFP) | 2016 | 3,9 | 3,00E-01 | Fülöp | 10.1364/OPTICA.3.001075 |
| 36 | Organic | 2018 | 1,1 | 0,26 | Rovere | 10.1364/OE.26.002509 |
| 37 | LN (TPFP) | 2018 | 200 | 0,3 | Wu | 10.1364/OE.26.007107 |
| 38 | GaP | 2018 |  | 2,72E-05 | Xu | 10.1088/1361-6455/aacfe7 |
| 39 | GaP | 2019 |  | 1,50E-03 | Drs | 10.1364/JOSAB.36.003039 |
| 40 | GaP | 2019 | 1,01E-04 | 1,20E-03 | Meyer | 10.1364/OE.27.030340 |
| 41 | LN (TPFP) | 2020 | 1,44E+00 | 4,20E-02 | Kramer | 10.1364/OE.389653 |
| 42 | Organic | 2020 | - | 2,00E-02 | Novelli | 10.3390/ma13061311 |
| 43 | GaP | 2020 | 6,00E-05 | 8,40E-06 | Hekmat | 10.1364/OME.402564 |
| 44 | Organic | 2021 | 116 | 1,5 | Gollner | 10.1063/5.0037235 |
| 45 | Organic | 2021 | 50 | 5,7 | Gollner | 10.1063/5.0037235 |
| 46 | LN (TPFP) | 2021 | 1400 | 7,00E-01 | Zhang | 10.1002/lpor.202000295 |
| 47 | LN (TPFP) | 2021 | 2,95 | 1,30E+00 | Guiramand | 10.1364/PRJ.428418 |
| 48 | Organic | 2022 | 17 | 2,50E+00 | Ovchinnikov | 10.1364/OL.475960 |

**Table** S1. References for Fig.1

**(b)**

Fig. S1 Achieved THz energy from the past 20 years (a), zoomed window for better visibility (b) with the references according to Table S1.

**(b)**

**(a)**

Fig. S2. Achieved THz generation efficiency from the past 20 years (a), zoomed window for better visibility (b) with the references according to Table S1.

1. **Parameters and references for calculating the curves in Figure 2.**

**GaP:**

$d_{eff}(\Omega)$ was calculated from $d_{eff}=\frac{\left| \chi^{2} \right|}{2}$, where $\chi^{2}(\Omega)$ was get from [S1]. Faust-Henry coefficient, $C_{1}=0.5$ for calculating $\chi^{2}$ was chosen as the average value from [S2] and [S3]. According to [S4], $r_{41}=0.97\frac{\mathrm{pm}}{V}$ @ 632.8 nm, thus $d_{41}=29\frac{pm}{V}$ and accordingly the nonlinear coefficient for the used polarization direction is $d_{eff}=33.5 pm/V$ $\left( \mathrm{since}: d_{eff}=\frac{2}{\sqrt{3}}d_{41} \right)$. For this, $\chi^{2}$ was set to have $d_{eff}=33.5\frac{pm}{V}$ close to 0 THz. Lorentz-model for optical frequencies was get from [S5], page 441, equation (1). For the THz-range, the oscillator frequency was got from [S3], and the damping constant from [S6] based on experimental values. $\epsilon_{\infty}=9.11$, and $\epsilon_{DC}=11.11$ were from [S7], page 101.

**GaAs:**

$d_{eff}$ was calculated similarly like GaP from $\chi^{2}(\Omega)$ from [S1], where all the Faust-Henry coefficients were also given. According to [S8]: $r_{41}=1.58\pm0.08\frac{\mathrm{pm}}{V} @ 1.15 \mu m$, thus $d_{41}=55.45\frac{pm}{V}$ and accordingly the nonlinear coefficient for the used polarization direction is $d_{eff}=64.0 pm/V$. For this, $\chi^{2}$ was set to have $d_{eff}=64.0\frac{pm}{V}$ close to 0 THz. For the optical frequencies, the Sellmeier-equation given with parameters in [S9] was used. Lorentz-model parameters for the THz frequencies was got from [S10], page 98.

**ZnTe:**

$d_{eff}$ was calculated similarly like GaP from $\chi^{2}\left( \Omega\right)$ from [S1], with Faust-Henry coefficients from [S3]. According to [S11]: $r_{41}=3.93\frac{\mathrm{pm}}{V} @ 1.04 \mu m$, thus $d_{41}=55.8 \frac{pm}{V}$ and accordingly the nonlinear coefficient for the used polarization direction is $d_{eff}=64.4 pm/V$. For this, $\chi^{2}$ was set to have $d_{eff}=64.4\frac{pm}{V}$ close to 0 THz. For the optical frequencies, the Sellmeier-equation given with parameters in [S12] was used. Lorentz-model parameters for the THz frequencies was got from [S13].

**LN:**

The parameters and the formula from [S14] was used to calculate $d_{eff}(\Omega)$. The parameters of the second oscillator term was changed to have a better fit to experimental values of [S15]. For room temperature (RT), $S_{2}=16.57;\gamma_{2}=0.93$ were used. At 100 K (CT) the amplitude parameter of the second oscillator was changed to $S_{2}=15.35\boldsymbol{,}$ and all the damping constants were multiplied by 0.55, based on [S16], where the ratio between damping constants at 80 K and 300 K was 25/62=0.4. The temperature dependent Sellmeier formula from [S17] was used for the optical properties. For the THz-range, the Lorentz-model in [S14] was used.

1. **Method to determine multiphoton absorption in the Section „High energy semiconductor-based terahertz sources”**

Table S2 contains the different order multiphoton absorption coefficients for GaP which was used to calculate the free carrier density in Fig. 6. Two- and six-photon absorption coefficients were used according to measured data [S18]. The other coefficients were calculated based on the Keldysh photoionization theory, according to which the ratio of the neighbor orders is approximately constant [S19].

| $\beta_{2}$  $\left( cm/GW \right)$ | $\beta_{3}$  $\left( \mathrm{cm}^{3}/\mathrm{GW}^{2} \right)$ | $\beta_{4}$  $\left( \mathrm{cm}^{5}/\mathrm{GW}^{3} \right)$ | $\beta_{5}$  $\left( \mathrm{cm}^{7}/\mathrm{GW}^{4} \right)$ | $\beta_{6}$  $\left( \mathrm{cm}^{9}/\mathrm{GW}^{5} \right)$ | $\beta_{7}$  $\left( \mathrm{cm}^{11}/\mathrm{GW}^{7} \right)$ |
| --- | --- | --- | --- | --- | --- |
| $\boldsymbol{4.0}$ | $1.22\cdot{10}^{-2}$ | $3.7\cdot{10}^{-5}$ | $1.14\cdot{10}^{-7}$ | $\boldsymbol{3.5\cdot}\boldsymbol{10}^{\boldsymbol{-10}}$ | $1.07\cdot{10}^{-12}$ |

**Table** S2. Values of multiphoton absorption coefficients. The measured values are highlighted.

Free carrier density was determined according to

|  | $N_{c}=\frac{\beta_{n}}{nh\nu_{0}}\int_{-\infty}^{\infty} I_{p}^{n}\left( t \right)dt,$ |  |
| --- | --- | --- |

where *n* is the absorption order, *h* is the Planck constant, $\nu_{0}$ is the central frequency of the pump, $I_{p}(t)$ is the pump intensity.

The THz absorption of the GaP crystal was determined based on

|  | $\alpha\left( \Omega\right)=2\frac{\Omega}{c}\mathfrak{I}m\left( \sqrt{\varepsilon_{r0}\left( \Omega\right)+\varepsilon_{fc}\left( \Omega\right)} \right),$ |  |
| --- | --- | --- |

where $\Omega$ is the THz angular frequency, *c* is the speed of the light,$\varepsilon_{r0}\left( \Omega\right)$ is the dielectric permittivity of the GaP crystal without free carriers, and

|  | $\varepsilon_{fc}\left( \Omega\right)=-\frac{q^{2}}{\varepsilon_{0}m_{eff}}\frac{N_{eff}}{\Omega^{2}+i\frac{\Omega}{\tau_{sc}}}$ |  |
| --- | --- | --- |

is the permittivity attributed to free carriers, where $q=1.6\cdot{10}^{-19} C$ is the charge of the electron, $\varepsilon_{0}=8.854\cdot{10}^{-12}F/m$ is the vacuum permittivity, $m_{eff}=0.11\cdot9.109\cdot{10}^{-31}\mathrm{kg}$ is the effective mass of the electron, $\tau_{sc}=200 fs$ is the electron scattering time, and $N_{eff}=N_{c}/2$ is the effective free carrier density [S20].

S1. T. Dekorsy, V. A. Yakovlev, W. Seidel, M. Helm, and F. Keilmann, "Infrared-Phonon–Polariton Resonance of the Nonlinear Susceptibility in GaAs," Phys. Rev. Lett. **90**(5), 055508 (2003).

S2. W. L. Faust, C. H. Henry, and R. H. Eick, "Dispersion in the Nonlinear Susceptibility of GaP near the Reststrahl Band," Phys. Rev. **173**(3), 781–786 (1968).

S3. A. Leitenstorfer, S. Hunsche, J. Shah, M. C. Nuss, and W. H. Knox, "Detectors and sources for ultrabroadband electro-optic sampling: Experiment and theory," Appl. Phys. Lett. **74**(11), 1516–1518 (1999).

S4. D. F. Nelson and E. H. Turner, "Electro‐optic and Piezoelectric Coefficients and Refractive Index of Gallium Phosphide," J. Appl. Phys. **39**(7), 3337–3343 (1968).

S5. E. D. Palik, ed., *Handbook of Optical Constants of Solids* (Acad. Press, 1998).

S6. K. Saito, T. Tanabe, Y. Oyama, K. Suto, T. Kimura, and J. Nishizawa, "Terahertz-wave absorption in GaP crystals with different carrier densities," J. Phys. Chem. Solids **69**(2–3), 597–600 (2008).

S7. O. Madelung, *Semiconductors: Data Handbook*, 3rd edition (Springer Berlin Heidelberg, 2004).

S8. C. ‐A. Berseth, C. Wuethrich, and F. K. Reinhart, "The electro‐optic coefficients of GaAs: Measurements at 1.32 and 1.52 μm and study of their dispersion between 0.9 and 10 μm," J. Appl. Phys. **71**(6), 2821–2825 (1992).

S9. T. Skauli, P. S. Kuo, K. L. Vodopyanov, T. J. Pinguet, O. Levi, L. A. Eyres, J. S. Harris, M. M. Fejer, B. Gerard, L. Becouarn, and E. Lallier, "Improved dispersion relations for GaAs and applications to nonlinear optics," J. Appl. Phys. **94**(10), 6447–6455 (2003).

S10. A. R. Long and Institute of Physics, eds., *Physics of Semiconductors 2002: Proceedings of the 26th International Conference on the Physics of Semiconductors Held in Edinburgh, UK, 29 July-2 August 2002*, Conference Series / Institute of Physics No. 171 (Institute of Physics Pub, 2003).

S11. Q. Song, L. Chai, W. Liu, Q. Ma, Y. Li, C. Wang, and M. Hu, "Measuring effective electro-optic coefficient at 1040 nm by spectral intensity modulation with THz time-domain spectroscopy," Infrared Phys. Technol. **97**, 54–57 (2019).

S12. D. T. F. Marple, "Refractive Index of ZnSe, ZnTe, and CdTe," J. Appl. Phys. **35**(3), 539–542 (1964).

S13. K. Wynne and J. J. Carey, "An integrated description of terahertz generation through optical rectification, charge transfer, and current surge," Opt. Commun. **256**(4–6), 400–413 (2005).

S14. D. Jang, J. H. Sung, S. K. Lee, C. Kang, and K.-Y. Kim, "Generation of 0.7 mJ multicycle 15 THz radiation by phase-matched optical rectification in lithium niobate," Opt. Lett. **45**(13), 3617 (2020).

S15. X. Wu, C. Zhou, W. R. Huang, F. Ahr, and F. X. Kärtner, "Temperature dependent refractive index and absorption coefficient of congruent lithium niobate crystals in the terahertz range," Opt. Express **23**(23), 29729 (2015).

S16. A. S. Barker and R. Loudon, "Dielectric Properties and Optical Phonons in LiNbO_3_," Phys. Rev. **158**(2), 433–445 (1967).

S17. O. Gayer, Z. Sacks, E. Galun, and A. Arie, "Temperature and wavelength dependent refractive index equations for MgO-doped congruent and stoichiometric LiNbO3," Appl. Phys. B **91**(2), 343–348 (2008).

S18. V. Nathan, S. S. Mitra, and A. H. Guenther, "Review of multiphoton absorption in crystalline solids," J. Opt. Soc. Am. B **2**(2), 294 (1985).

S19. L. Keldysh, "Ionization in the field of a strong electromagnetic wave," Sov Phys JETP **20**(5), 1307–1314 (1965).

S20. N. M. Mbithi, G. Tóth, Z. Tibai, I. Benabdelghani, L. Nasi, G. Krizsán, J. Hebling, and G. Polónyi, "Investigation of terahertz pulse generation in semiconductors pumped at long infrared wavelengths," J. Opt. Soc. Am. B **39**(10), 2684 (2022).

1. † These authors contribute equally [↑](#footnote-ref-1)
2. [↑](#footnote-ref-2)
